# Weighing the Benefits and Risks of Medical Marijuana Use: A Brief Review

**DOI:** 10.3390/pharmacy6040128

**Published:** 2018-12-06

**Authors:** Allison Karst

**Affiliations:** PGY2 Psychiatric Pharmacy Resident, Veterans Affairs Tennessee Valley Healthcare System, Nashville, TN 37212, USA; allison.karst@va.gov; Tel.: +502-741-4979

**Keywords:** medical marijuana, marijuana, cannabis

## Abstract

Despite federal prohibition of medical marijuana possession, sale, and use, marijuana use continues to escalate as state legalization persists and expands. The purpose of this discussion is to provide a brief summary of the evidence regarding both potential benefits and risks of medical marijuana use.

Despite federal prohibition of medical marijuana possession, sale, and use, marijuana use continues to escalate as individual state legalization persists and expands (Table 1). As the medical marijuana landscape rapidly changes, it is imperative that healthcare providers stay up to date on available evidence regarding both the benefits and risks of use. Although it is important to note potential benefits demonstrated in specific disease states, evidence in most qualifying indications is insufficient, with the majority lacking randomized controlled trials (RCTs) (Table 2).

Marijuana and oral cannabinoids (dronabinol, nabilone, oral THC) have been associated with adverse effects, including serious adverse effects as well as study withdrawal. The most commonly reported adverse effects include asthenia, balance problems, disorientation, gastrointestinal effects, euphoria, somnolence, dry mouth, fatigue, hallucinations, paranoia, and agitation [11,17]. 

Marijuana use has a negative effect on mental health and neurologic function. Marijuana users are at risk for tolerance, dependence, and withdrawal [4,18,44]. Multiple studies have examined the negative effects of marijuana on acute and long-term cognition, including impairment in attention, impulse control, decision-making processes, working memory, and executive function. Additionally, marijuana has been associated with an early onset of psychotic disorders, an exacerbated course of illness in established psychotic disorders, exacerbation of mania in bipolar disorders, and worsened symptoms of PTSD [4,11,18,41,44].

Pulmonary, cardiovascular, and carcinogenic effects of marijuana remain controversial [4,44,48,49]. In vivo and in vitro studies have demonstrated that marijuana inhibits several hepatic enzymes (CYP2D6, CYP2C19, CYP2C9, CYP3A4), and preliminary evidence in humans suggests that marijuana may interact with serum drug concentrations of warfarin and antiretroviral therapies [16,50,51,52]. However, additional research is warranted in these areas as the risk of clinically significant drug interactions is unknown [53].

When discussing medical marijuana use, pharmacists must be knowledgeable about potential benefits and risks. Disease states with substantial evidence include chronic pain, chemotherapy-induced nausea and vomiting (oral cannabinoids only), and patient-reported spasticity in MS [2]. Further research is warranted, particularly regarding products similar to those currently available in dispensaries. Because medical marijuana lacks quality standards and FDA regulation, available products have shown significant inconsistencies, with one study revealing that only 17% of edible cannabis products were accurately labeled [54]. It is the responsibility of prescribers and pharmacists to educate patients on potential adverse events and drug interactions. Other pertinent issues to consider are marijuana dosing as well as the inability to extrapolate evidence between oral cannabinoids and marijuana due to differences in chemical composition. As a result of limited high-quality evidence and lack of regulation, the potential benefits and risks must be weighed carefully to make appropriate clinical decisions.

## Figures and Tables

**Table 1:** Qualifying indications by state [1].

State [1]																															
Qualifying Conditions	AK	AR	AZ	CA	CO	CT	DC	DE	FL	HI	IL	MA	MD	ME	MI	MN	MO	MT	ND	NH	NJ	NM	NV	NY	OH	OR	PA	RI	UT	VT	WA
Alzheimer’s Disease		X	X				X	X			X			X	X		X		X	X					X	X		X	X		
ALS (Lou Gehrig’s Disease)		X	X			X	X	X	X		X	X		X	X	X	X		X	X	X	X		X	X		X		X		
Autism							X										X										X		X		
Arthritis		X		X			X															X									
Cachexia (or Anorexia)	X	X	X	X	X	X	X	X		X	X		X	X	X	X	X	X	X	X		X	X			X		X	X	X	X
Cancer	X	X	X	X	X	X	X	X	X	X	X	X		X	X	X	X	X	X	X	X	X	X	X	X	X	X	X	X	X	X
Cerebral palsy						X	X																								
Chronic or Debilitating Disease		X				X	X				X			X			X		X	X	X	X		X			X				
Chronic pain	X	X	X	X	X	X	X	X	X	X	X		X	X	X	X	X	X	X	X		X	X	X	X	X	X	X	X	X	X
Crohn’s Disease		X	X			X	X		X	X		X		X	X	X	X	X	X	X		X			X		X	X	X	X	X
Cystic Fibrosis						X	X																								
Fibromyalgia		X			X		X				X								X						X						
Glaucoma	X	X	X	X	X	X	X		X	X	X	X		X	X	X	X	X	X	X	X	X	X		X	X	X	X		X	X
Hepatitis C		X	X				X				X	X		X	X		X		X	X		X			X			X			X
HIV/AIDS	X	X	X	X	X	X	X	X	X	X	X	X		X	X	X	X	X	X	X	X	X	X	X		X	X	X	X	X	X
Migraine				X			X										X														
Multiple Sclerosis	X	X				X	X		X	X	X	X		X			X		X	X	X	X		X	X		X		X	X	
Nausea	X	X	X	X	X		X	X		X	X		X	X	X			X	X	X		X	X			X		X		X	X
Parkinson’s Disease						X	X		X		X	X		X			X			X		X		X	X		X			X	
Persistent Muscle Spasms		X	X	X	X	X	X	X	X	X			X		X	X	X	X	X	X	X		X			X	X	X	X		X
Peripheral neuropathy		X			X		X				X						X					X		X			X				
Psoriasis						X	X																								
PTSD		X	X		X	X	X	X	X	X	X			X	X	X	X		X	X	X	X	X		X	X	X	X	X	X	X
Seizures (or Epilepsy)	X	X	X	X	X	X	X	X	X	X	X		X	X	X	X	X	X	X	X	X	X	X	X	X	X	X	X	X	X	X
Terminal Illness							X	X	X							X	X				X	X					X			X	X
Tourette’s syndrome		X					X				X						X								X						
Ulcerative Colitis		X				X	X							X											X						

**Table 2:** Potential benefits of marijuana [1,2,3,4,5,6,7,8,9,10,11,12,13,14,15,16,17,18,19,20,21,22,23,24,25,26,27,28,29,30,31,32,33].

Qualifying Condition	Summary/Quality of Evidence
**Amyotrophic Lateral Sclerosis (ALS) [2,3]**	**Insufficient evidence**; Single RCT showed no significant difference on primary or secondary outcomes
**Alzheimer’s Disease [2,4,5,6,7,8,9]**	No published RCTs. Cochrane systematic review concluded that there is **insufficient evidence** that oral cannabinoids are effective for the treatment of behavioral disturbances associated with dementia. There is **limited evidence** that oral cannabinoids are **ineffective**.
**Autism [10]**	Data limited to animal model-based research, small case series or single case reports
**Arthritis [11]**	While there is moderate evidence in chronic pain, there is **insufficient evidence** in arthritic conditions
**Cachexia/Anorexia [2,11,12,13,14,15]**	U.S. Food and Drug Administration (FDA)-approved product available (dronabinol); low-quality evidence
**Cancer [16]**	RCTs only in supportive care measures; preliminary evidence of anti-proliferative properties indicates an area of future research
**Cerebral Palsy [2]**	Low to moderate evidence for symptoms of cerebral palsy, such as pain, spasms, and seizures; however, there is **insufficient evidence** in this specific population for recommendations
**Chronic Pain [2,11,17,18]**	Substantial evidence for benefit with cannabis; moderate evidence for benefit with nabiximols
**Crohn’s Disease [19,20]**	**Insufficient and inconclusive evidence**
**Cystic Fibrosis [2]**	Preliminary evidence only; No RCTs; **insufficient evidence**
**Fibromyalgia [21,22]**	Cohort studies only, no RCTs for pain in fibromyalgia; **moderate evidence in sleep improvement**
**Glaucoma [2,23,24,25,26,27]**	Significantly lowers intraocular pressure (IOP) for 4 hours; **Insufficient evidence** to indicate that marijuana is safer or more effective than existing pharmacotherapy or surgery for reduction of IOP
**Hepatitis C [2]**	No RCTs; **insufficient evidence**
**Human Immunodeficiency Virus (HIV)/Acquired Immunodeficiency Syndrome (AIDS) [28,29,30,31]**	Moderate evidence for FDA-approved product (dronabinol); Low-quality evidence suggesting that cannabinoids and marijuana are associated with weight gain in patients with HIV/AIDS
**Migraine [32]**	Retrospective chart review demonstrated decreased frequency of migraines; No RCTs; **insufficient evidence**
**Multiple Sclerosis (MS) or Persistent Muscle Spasms [2,33,34,35,36]**	Moderate quality evidence to suggest benefit
**Nausea [2,17,37]**	FDA-approved products available (nabilone, dronabinol); Low quality evidence for benefit with marijuana
**Parkinson’s Disease [38,39,40]**	Results of trials studying the use of oral cannabinoids in Parkinson’s Disease have been **controversial and inconclusive**. Most importantly, there have been **no RCTs** examining marijuana specifically in this population.
**Peripheral Neuropathy [2,11,18]**	**Moderate to substantial evidence** to suggest benefit of cannabinoids in peripheral neuropathy
**Psoriasis [2]**	No RCTs; **insufficient evidence**
**Post-Traumatic Stress Disorder (PTSD) [41,42,43,44]**	Insufficient evidence; potential harm
**Seizures [45,46,47]**	Moderate-quality evidence for use of cannabidiol (CBD) (but not marijuana) as adjunctive therapy in patients with refractory seizures
**Tourette’s Syndrome [10]**	**Limited evidence** that ∆-9-tetrahydrocannabinol (THC) capsules are an effective treatment for improving symptoms of Tourette syndrome
**Ulcerative Colitis [2]**	No RCTs; **insufficient evidence**
